# Bone marrow stromal cells from low-turnover osteoporotic mouse model are less sensitive to the osteogenic effects of fluvastatin

**DOI:** 10.1371/journal.pone.0202857

**Published:** 2018-08-24

**Authors:** Yukari Oda, Hodaka Sasaki, Tadashi Miura, Takuya Takanashi, Yoshitaka Furuya, Masao Yoshinari, Yasutomo Yajima

**Affiliations:** 1 Oral Health Science Center, Tokyo Dental College, Tokyo, Japan; 2 Department of Oral and Maxillofacial Implantology, Tokyo Dental College, Tokyo, Japan; Mayo Clinic Minnesota, UNITED STATES

## Abstract

This study aimed to investigate the effects of fluvastatin on the differentiation of bone marrow stromal cells (BMSCs) into osteoblasts in senescence-accelerated mouse prone 6 (SAMP6) compared with that in the normal senescence-accelerated-resistant mouse (SAMR1) model. SAMP strains arose spontaneously from the AKR/J background and display shortened life span and an array of signs of accelerated aging, compared with control SAMR strains. The dose effects of fluvastatin were also evaluated. BMSCs were cultured with/without fluvastatin (0 μM, 0.1 μM, 0.5 μM, and 1.0 μM). WST-1-based colorimetry was performed to evaluate cell proliferation. To evaluate cell differentiation, gene expression levels of bmp2 and runx2 were determined by quantitative reverse transcription polymerase chain reaction (qRT-PCR), and protein expression levels were determined using enzyme-linked immunosorbent assay (BMP2) and immunofluorescence staining (BMP2 and Runx2). Alkaline phosphatase (ALP) activity assay and histochemical detection were determined; the effect of noggin, a BMP-specific antagonist, was examined using ALP histochemical detection. To assess for mature osteogenic marker, gene expression levels of bglap2 were determined by qRT-PCR and mineralization was determined by alizarin red staining. RhoA activity was also examined by Western blotting. In SAMP6, BMP2, Runx2 and Bglap2 mRNA and protein expressions were significantly increased by fluvastatin, and ALP activity was increased by BMP2 action. RhoA activity was also inhibited by fluvastatin. The concentration of fluvastatin sufficient to increase BMP2 and Runx2 expression and ALP activity was 0.5 μM in SAMP6 and 0.1 μM in SAMR1. In conclusion, the present study revealed that fluvastatin promoted BMSC differentiation into osteoblasts by RhoA-BMP2 pathway in SAMP6. BMSCs of SAMP6 are less sensitive to the osteogenic effects of fluvastatin than SAMR1.

## Introduction

Dental implant treatment is widely used. In aging societies, the number of elderly patients who receive implant treatment is increasing [1]. Osteoporosis, including high-turnover and low-turnover osteoporosis, is a well-known risk factor for the prognosis of dental implants [2]. In high-turnover osteoporosis, bone resorption increases because of estrogen deficiency; in low-turnover osteoporosis, the ossification potential is compromised because of aging [3]. Low-turnover osteoporosis is observed not only in women but also in men [3]. Several studies regarding high-turnover osteoporosis have been performed, whereas studies regarding low-turnover osteoporosis are scarce. Therefore, investigation of low-turnover osteoporosis is important for treatment of elderly people.

Low-turnover osteoporosis causes both cortical and trabecular bone reduction [4]. SAMP (senescence-accelerated mouse prone) strains arose spontaneously from the AKR/J background and display shortened life span and an array of signs of accelerated aging, compared with control SAMR strains [5]. Several studies regarding low-turnover osteoporosis have used SAMP6 mouse strain, which exhibits low-turnover osteoporosis [6]. In particular, this strain is characterized by decreased osteoblastogenesis, resulting in decreased bone formation and delayed bone healing [7]. Furthermore, it has been reported that peri-implant bone density in SAMP6 is low during osseointegration [8]. Therefore, improvement of bone healing in low-turnover osteoporosis is required.

Statins are competitive inhibitors of 3-hydroxy-2-methylglutaryl coenzyme A (HMG-CoA) reductase and are widely used to lower cholesterol levels in patients with hyperlipidemia and arteriosclerosis [9]. Another effect of statins has recently been reported in several studies: statins stimulate the differentiation of osteoblasts through BMP2, indicating their potential in the development of new osteogenic drugs [10,11]. In previous animal experiments, the effects of fluvastatin in normal animal models and osteoporosis animal models have been investigated. In normal animal models, local administration of simvastatin improves fracture healing in the rat femur [12]. In studies using low-turnover osteoporosis models, it was reported that local administration of a fluvastatin improved bone healing in SAMP6 [13]. It was also reported that improvement of bone volume by local administration of fluvastatin was lower in SAMP6 than in SAMR1. Accordingly, further studies are required to clarify the fluvastatin doses that are sufficient for obtaining better bone healing.

No *in vitro* studies have been reported using a low-turnover osteoporosis mouse model. In addition, the dose effects of fluvastatin on bone marrow stromal cell (BMSC) differentiation into osteoblasts and the function of fluvastatin in BMSC differentiation are yet to be elucidated.

Therefore, this study aimed to investigate the effect of fluvastatin on BMSC differentiation into osteoblasts in SAMP6 as a low-turnover osteoporosis model compared with SAMR1 as a normal model. In addition, the dose effects of fluvastatin were evaluated.

## Materials and methods

### Fluvastatin

Fluvastatin sodium salt (Toronto Research Chemicals Inc., Ontario, Canada) was used in various concentrations: 0 μM (as control, MillQ water), 0.1 μM, 0.5 μM, and 1.0 μM dissolved in MilliQ and filter-sterilized to evaluate the dose effect of fluvastatin.

### Animals

Twenty-week-old male SAMP6 mice (Japan SLC Inc., Shizuoka, Japan) and SAMR1 mice (Japan SLC Inc.) were used as models of low-turnover osteoporosis models and controls, respectively. Animal experiments in this study were conducted in strict accordance with the Tokyo Dental College’s IACUC committee Guidelines for Animal Experiments (Approval protocol number: 253006, Approval date: 4/1/2013, Title: Effects of fluvastatin on bone marrow stromal cells of senescence-accelerated mouse prone 6). Animals were housed under standard husbandry conditions and given access to food and water. Daily care was performed by husbandry staff.

### Preparation and culture of BMSCs

SAMP6 mice (n = 3) and SAMR1 mice (n = 3) were euthanized under deep anesthesia with intraperitoneal sodium pentobarbital (Somnopentyl, Kyoritsu Seiyaku Co. Ltd., Tokyo, Japan). Metaphyses of femurs and tibias were aseptically cut and diaphysis cavities were flushed with culture medium. Culture medium was a minimum essential medium alpha (αMEM; Gibco by Life technologies, Grand Island, NY, USA), containing 15% fetal bovine serum (FBS; biowest, Nuaillé, France), 100 U/mL penicillin (Gibco by Life technologies, Grand Island, NY USA) and 100 μg/mL streptomycin (Gibco by Life technologies, Grand Island, NY USA); cell cultures were maintained at 37°C in an atmosphere of 5% CO_2_ and 100% humidity. Bone marrow cells were subsequently collected and plated, and after 24 h, red blood cells and non-adherent cells were removed. After reaching 80% confluence, cells were trypsinated and plated as the first passage of the culture. Culture medium was subsequently changed to osteogenic differentiation medium (αMEM supplemented with 50 μg/mL ascorbic acid and 10 mM β-glycerolphosphate; all from Sigma, St. Louis, MO, USA) and replaced every 3 days. All tests were performed on the first passage, and cells were seeded at a density of 2.0 × 10^4^ cells/cm^2^ and mixed with various concentration of fluvastatin as mentioned above.

### Cell proliferation assay

Cells were seeded in 96-well plates. Cell proliferation was tested using WST-1-based colorimetry (Roche Applied Science, Mannheim, Germany). After 1, 3, 7, and 14 days of culture, WST-1 was added to each well and incubated at 37°C for 1 h (n = 5). After incubation, the resulting supernatants were measured for absorbance at 450 nm and recorded using a microplate reader (SpectraMax M5, Molecular Devices, Sunnyvale, CA, USA).

### Quantitative real-time polymerase chain reaction

Expression levels of *bmp2*, *runx2* and *bglap2* were determined by a quantitative real-time reverse transcription polymerase chain reaction (qRT-PCR) assay, and cells obtained after 1, 3, 7, and 14 days of culture were used (n = 5). The list of genes and primers are shown in Table 1. In *bglap2* experiments, cells after 21days culture were used. Data was normalized to the control gene *gapdh*, evaluated by relative mRNA abundance as determined by the ΔΔCt method, and reported as fold induction. A commercial kit (RNeasy Mini kit, QIAGEN, Germany) was used to extract the mRNA from the cell pellets, and quantification of RNA was performed by measuring absorbance with the Nano-Drop^®^ Spectrophotometer ND-1000 using the ND-1000 software version 3.5.2 (Thermo Scientific, West Palm Beach, FL, USA). Total RNA was reverse-transcribed using a commercial kit (ReverTraAce^®^ qPCR RT Master Mix, Toyobo, Osaka, Japan), and qRT-PCR was performed with the THUNDERBIRD^®^ Probe qPCR Mix (Toyobo, Osaka, Japan) with standardized primers (TaqMan^®^ Gene Expression Assays, Applied Biosystems by Life technologies, NY, USA). Samples were analyzed using the Applied Biosystems 7500 Real-Time PCR system with SDS software version 1.4.

**Table 1:** ABI Taqman gene expression assay.

Gene symbol	LocusLink gene name	ABI assay ID
*GAPDH*	glyceraldehyde-3-phosphate dehydrogenase	Mm99999915_g1
*bmp2*	bone morphogenetic protein 2	Mm01962382_s1
*runx2*	runt-related transcription factor 2	Mm00501584_m1
*bglap2*	bone gamma carboxyglutamate protein	Mm03413826_mH

### Enzyme linked immunosorbent assay analysis

After 3, 7, and 14 days of fluvastatin treatment, the BMP2 concentration in cell lysates was measured using BMP2 Mouse enzyme linked immunosorbent assay (ELISA) kit (ab119582, Abcam, Cambridge, UK) according to the manufacturer’s instructions. BMP2 concentrations were normalized to protein content. The protein content was determined using a BCA protein assay reagent (BCA, PierceChemical, Rockford, IL, USA) (n = 5).

### Immunofluorescence staining

For immunofluorescence experiments, cells were plated onto glass coverslips. After fluvastatin treatment, cells were fixed in 10% neutral buffered formalin. Cells were incubated with a primary antibody against BMP2 (1:100) (Bioworld Technology Inc., MN, USA) and Runx2 (1:100) (ab76956, Abcam, Cambridge, UK). Then, the samples were incubated with the appropriate secondary antibody, 4ʹ,6-diamidino-2-phenylindole (DAPI), and phalloidin. Coverslips mounted in an antifading mounting medium (ProLong^®^ Gold antifade reagent; Invitrogen, Eugene, OR, USA) were examined by CSLM using a LSM5 DUO microscope (Carl Zeiss MicroImaging, Gőttingen, Germany) with a 63× oil immersion objective. Images were analyzed using ZEN 2008 software (Carl Zeiss). To evaluate BMP2 positivity, the numbers of stained (positive) and unstained (negative) cells were counted. The data were expressed as the mean percentage of the ratio of the number of positive cells relative to the total number of cells. (n = 5).

### Alkaline phosphatase activity and mineralization

Alkaline phosphatase (ALP) activity was measured using a commercially available kit (LabAssay^™^ ALP kit, Wako Pure Chemicals, Tokyo, Japan) according to standard procedures. After 3, 7, and 14 days of fluvastatin treatment, the samples were subsequently detached using a cell scraper and sonicated on ice (Branson, MO, USA) (n = 5). Cell debris was removed by centrifugation at 15,000 rpm. ALP activity levels were normalized against total protein using a protein assay reagent (BCA, Pierce Chemical, Rockford, IL, USA). After 14 days of fluvastatin treatment, cells were fixed in 10% neutral buffered formalin. ALP substrate solution (Roche Diagnostics, Basel, Switzerland) was added to fixed cells for staining. Cells were subsequently washed with PBS and images were recorded.

After 21 days of fluvastatin treatment, cells were fixed in 10% neutral buffered formalin and then stained with Alizarin Red S solution (Wako Pure Chemical Industries Ltd., Osaka, Japan) for 5 min at room temperature. Cells were washed with PBS and images were recorded.

### BMP inhibition (noggin) assay

A total of 250 ng/mL recombinant mouse noggin (R&D Systems, Minneapolis, MN, USA) was applied to cells in the absence or presence of fluvastatin (SAMR1 at 0.1 μM and SAMP6 at 0.5 μM). On day 14, the cells were harvested, and we proceeded with ALP staining as mentioned above. (n = 5).

### RhoA activation assay

RhoA activity was assayed using rhotekin beads. After 3days of fluvastatin culture, Rho Activation Assay Kit (Cytoskelton, Denver, USA) was applied following manufacturer’s instructions. Cells were lysed using lysis buffer, and the cell lysates were clarified by centrifugation, before incubation with Rho Assay Reagent to selectively bind GTP-RhoA and RhoA in a pull-down assay. Whole-cell lysates were immunoblotted directly to determine the total amount of RhoA, where RhoA was detected by Western blotting [14]. In studies of inhibitors of statin-induced RhoA inactivation, cells were treated with 1 mM mevalonate (Sigma, St. Louis, MO) in the presence and absence of fluvastatin.

### Statistical analysis

Results were expressed as means ± standard deviations. Statistical analysis was performed using GraphPad Prism (version 6.0; GraphPad Software Inc., San Diego, CA, USA). The data were analyzed by one-way analysis of variance (ANOVA), followed by Tukey’s multiple comparison test, and probability (*p*) values <0.05 were considered statistically significant. In mineralization and RhoA activation assay, Student’s *t*-test was used.

## Results

### Effects of fluvastatin on cell proliferation

**Fig 1:**
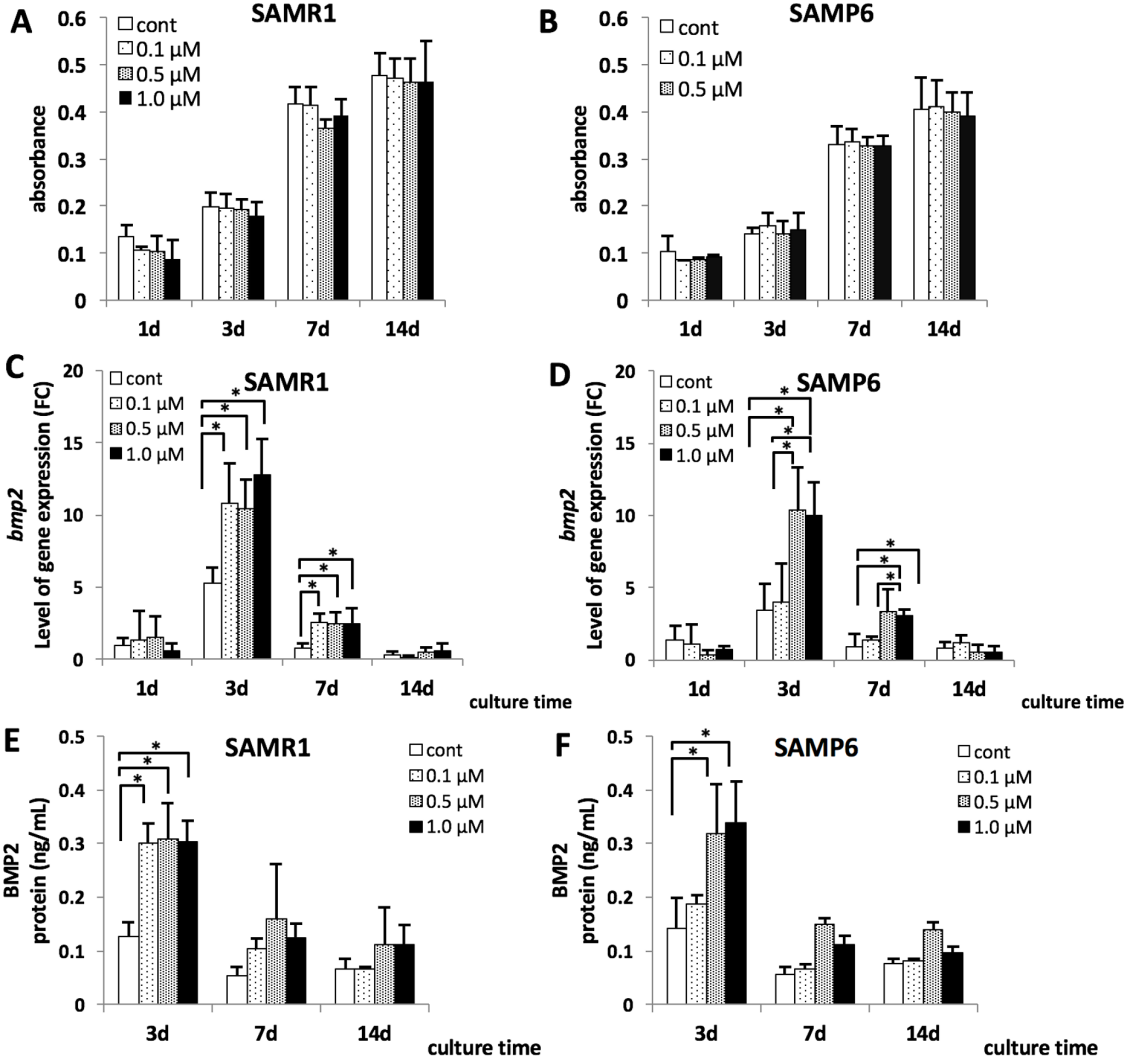
Cell proliferation of SAMR1 and SAMP6 BMSCs (A, B), *bmp2* mRNA expression in SAMR1 and SAMP6 BMSCs (C, D) and the concentrations of BMP2 examined using ELISA (E, F). (A, B) The cells were treated with 0 μM, 0.1 μM, 0.5 μM and 1.0 μM of fluvastatin for 1, 3, 7 and 14 days, and cell proliferation was assessed using WST-1-based colorimetory. No significant differences in cell proliferation were associated with differences in concentrations of fluvastatin in SAMR1 and SAMP6 on any day. (C, D) The expression level of *bmp2* mRNA was shown as FC (fold chnage). (ΔΔCt method, baseline = 1day on control in SAMR1) *P < 0.05 *bmp2* mRNA expression was significantly increased at fluvastatin concentrations more than 0.1 μM at 3 and 7 days of culture in SAMR1, and at concentrations more than 0.5 μM at 3and 7 days in SAMP6. (E, F) In ELISA assay, BMP2 concentrations were significantly increased at concentrations more than 0.1 μM at 3 days of culture in SAMR1, and at concentrations more than 0.5 μM at 3 days of culture in SAMP6.

Cell proliferation in SAMP6 tended to be decreased compared with that in SAMR1. No significant differences in cell proliferation were associated with differences in concentrations of fluvastatin in SAMR1 and SAMP6 on any day (Fig 1A and 1B).

### Effects of fluvastatin on BMP2 mRNA and protein expression

Effects of fluvastatin on *bmp2* mRNA and BMP2 protein expression are shown in Fig 1A, 1B, 1C and 1D respectively. In SAMR1 BMSCs, *bmp2* mRNA expression was significantly increased at fluvastatin concentrations more than 0.1 μM at 3 days and 7 days of culture (Fig 1C), whereas *bmp2* mRNA expression in SAMP6 BMSCs was significantly increased at concentrations >0.5 μM at 3 days and 7 days of culture (Fig 1D). In the protein assay, ELISA analysis showed that BMP2 concentrations in SAMR1 BMSCs were significantly increased at concentrations >0.1 μM at 3 days of culture (Fig 1E). In SAMP6 BMSCs, the BMP2 concentration was significantly increased at concentrations >0.5 μM at 3 days of culture (Fig 1F).

**Fig 2:**
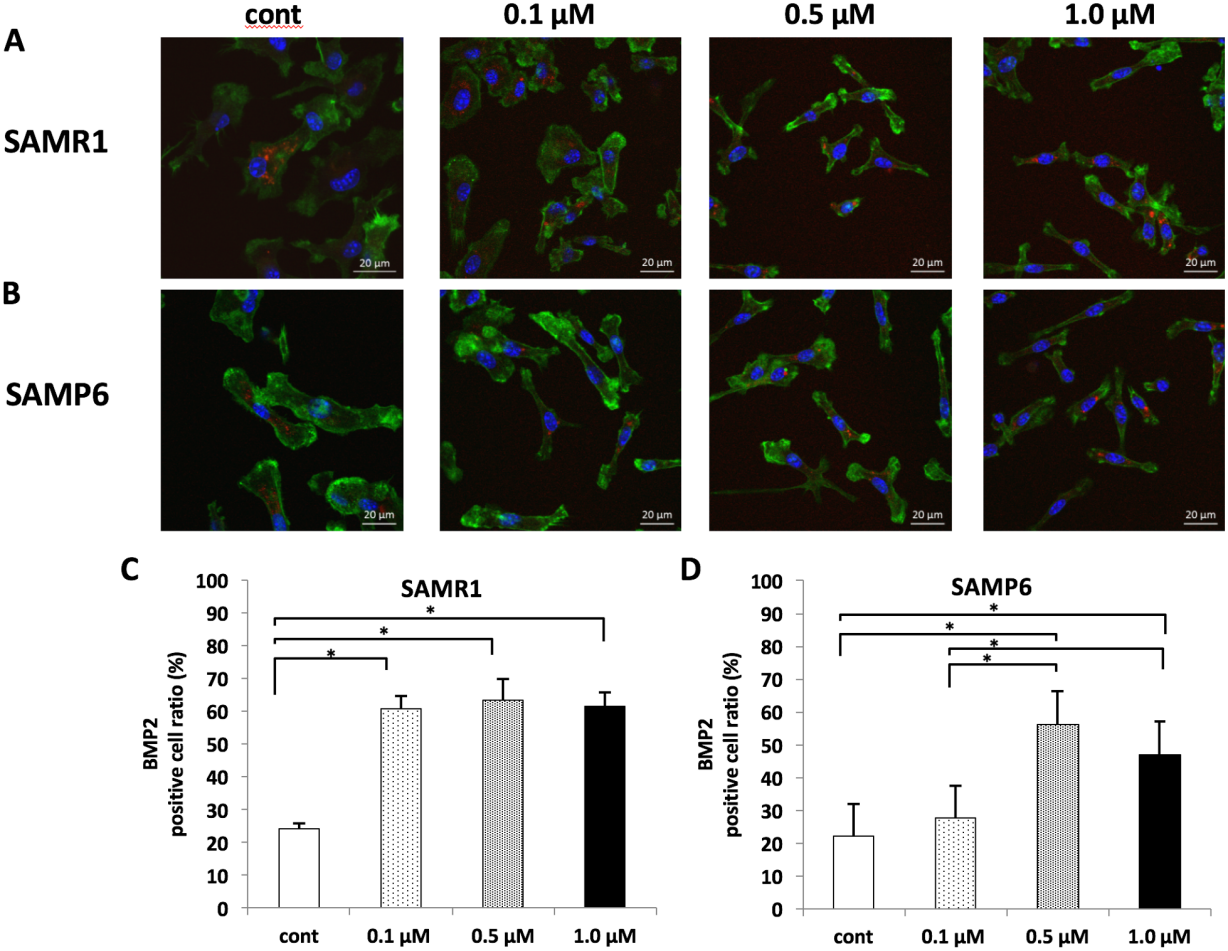
The protein expression of BMP2 detected with immunofluorescence staining (A, B) and percentages of BMP2 positive cells for 3days fluvastatin treatment in immunofluorescence staining (C, D). (A, B) BMP2 expressions were found in the cytoplasm. Red: BMP2, Blue: DAPI and green: actin. Scale bar = 20 μm. (C, D) The percentage of BMP2-positive stained cells was significantly higher at concentrations more than 0.1 μM in SAMR1 and at concentrations more than 0.5 μM in SAMP6. *P < 0.05.

Using immunofluorescence staining, a BMP2-positive reaction was observed at all concentrations in SAMR1 BMSCs (Fig 2A). In SAMP6 BMSCs, a BMP2-positive reaction was also observed at all concentrations (Fig 2B). In addition, the percentage of BMP2-positive stained cells was significantly higher at concentrations >0.1 μM in SAMR1 (Fig 2C), whereas in SAMP6, it was significantly higher at concentrations >0.5 μM than in controls and at 0.1 μM (Fig 2D).

### Effects of fluvastatin on Runx2 mRNA and protein expression

**Fig 3:**
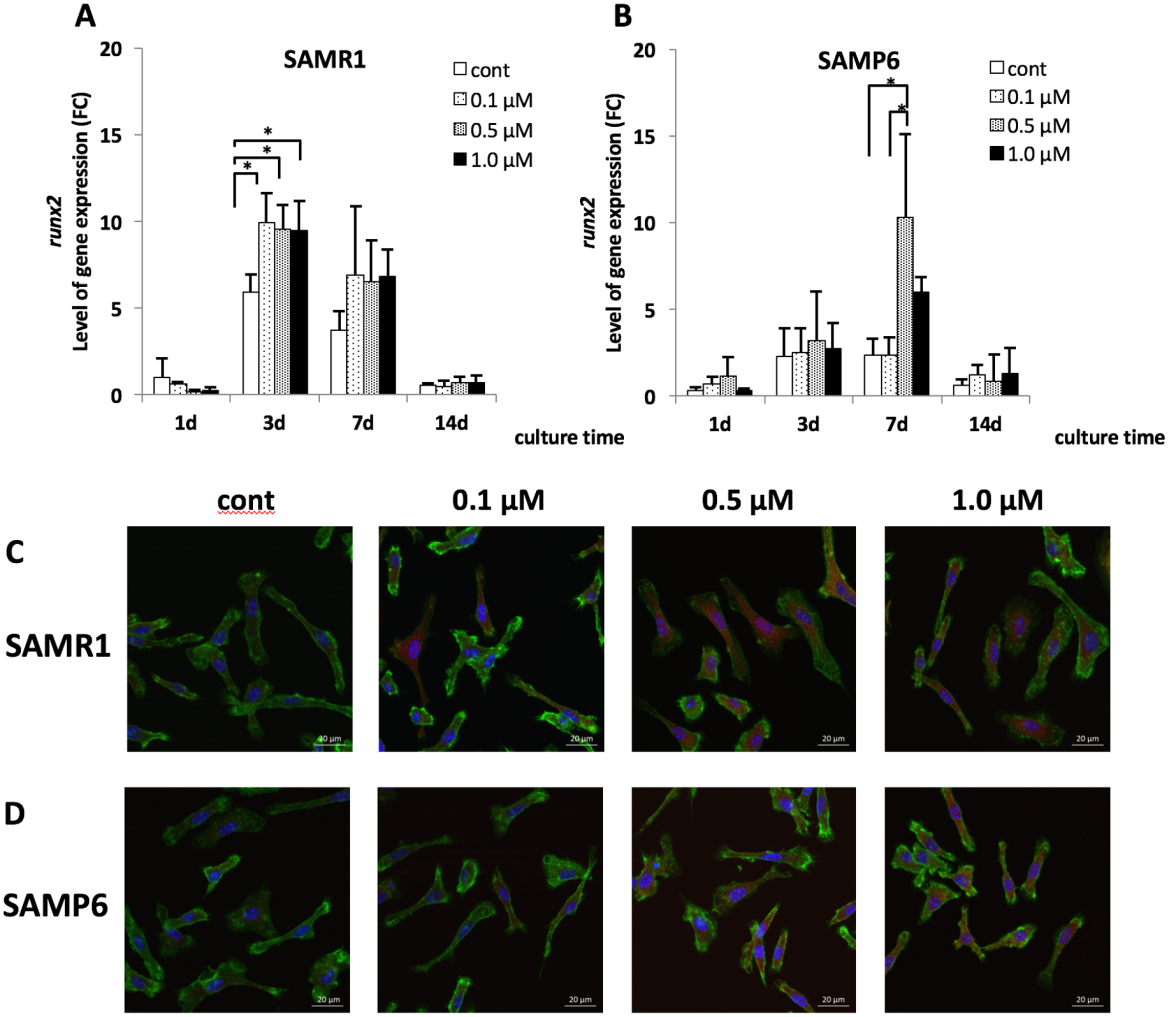
*runx2* mRNA expression (A, B) and protein expression of Runx2 (C, D) in SAMR1 and SAMP6 BMSCs. (A, B) The expression level of *runx2* mRNA was shown as FC (fold chnage). (ΔΔCt method, baseline = 1day on control in SAMR1) *P < 0.05 *runx2* mRNA expression was significantly increased at 0.1μM, 0.5μM and 1.0μM fluvastatin at 3 days of culture in SAMR1, and at 0.5 μM at 7 days in SAMP6. (C, D) BMSCs were treated with fluvastatin for 7days. Red: Runx2, blue: DAPI and green: actin. Scale bar = 20 μm.

Effects of fluvastatin on *runx2* mRNA and protein expression detected with immunofluorescence staining are shown in Fig 3. The *runx2* mRNA expression in SAMR1 BMSCs treated with 0.1 μM, 0.5 μM, and 1.0 μM fluvastatin significantly increased compared with that in controls at 3 days (Fig 3A), whereas *runx2* expression significantly increased in SAMP6 BMSCs treated with 0.5 μM fluvastatin compared with those treated with 0 μM and 0.1 μM fluvastatin at 7 days (Fig 3B). In addition, Runx2 protein expression in SAMR1 BMSCs was highly observed at more than 0.1 μM fluvastatin, and that in SAMP6 BMSCs was highly observed at more than 0.5 μM (Fig 3C and 3D).

### Effects of fluvastatin on ALP activity and histochemical detection

**Fig 4:**
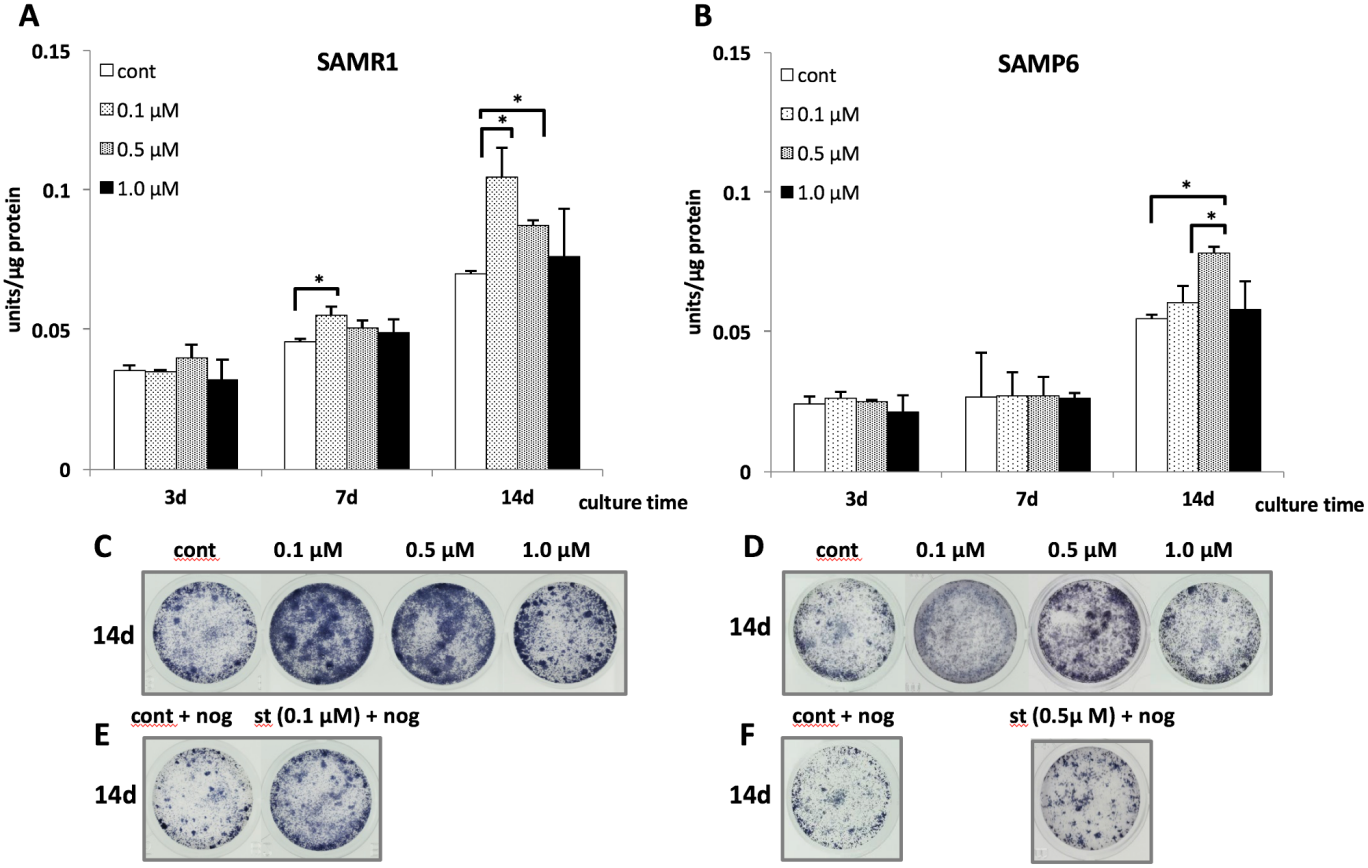
The activity of intracellular alkaline phosphatase (ALP) and histochemical detection of ALP in SAMR1 (A, C, E) and SAMP6 (B, D, F) BMSCs. (A, B) ALP activity was normalized by total cellular protein. (C, D) Histochemical detection of ALP in SAMR1 and SAMP6 BMSCs for 14days treatment of fluvastatin. (E, F) SAMR1 and SAMP6 BMSCs were treated for 14days in the presence or absence of fluvastatin (SAMR1 at 0.1μM and SAMP6 at 0.5μM) and noggin (250 ng/ml). *P < 0.05.

In SAMR1 BMSCs, the ALP activity at a concentration of 0.1 μM fluvastatin was higher than that in controls at 7 days, and this effect was observed at concentrations of 0.1 μM and 0.5 μM fluvastatin compared with control at 14 days (Fig 4A). In SAMP6 BMSCs, ALP activity at a concentration of 0.5 μM fluvastatin was higher than that of the control and at 0.1 μM at 14 days (Fig 4B). Histochemical detection of ALP activity at 14 days showed similar phenomena for ALP activity in both SAMR1 and SAMP6 BMSCs (Fig 4C and 4D).

### Effects of noggin on fluvastatin-induced ALP activity

Effects of noggin, a BMP-specific antagonist, on fluvastatin-induced ALP evidenced by histochemical analysis are shown in Fig 4E and 4F. Noggin treatment inhibited fluvastatin-induced ALP activity in both SAMP6 and SAMR1 BMSCs.

### Effects of fluvastatin on mineralization

**Fig 5:**
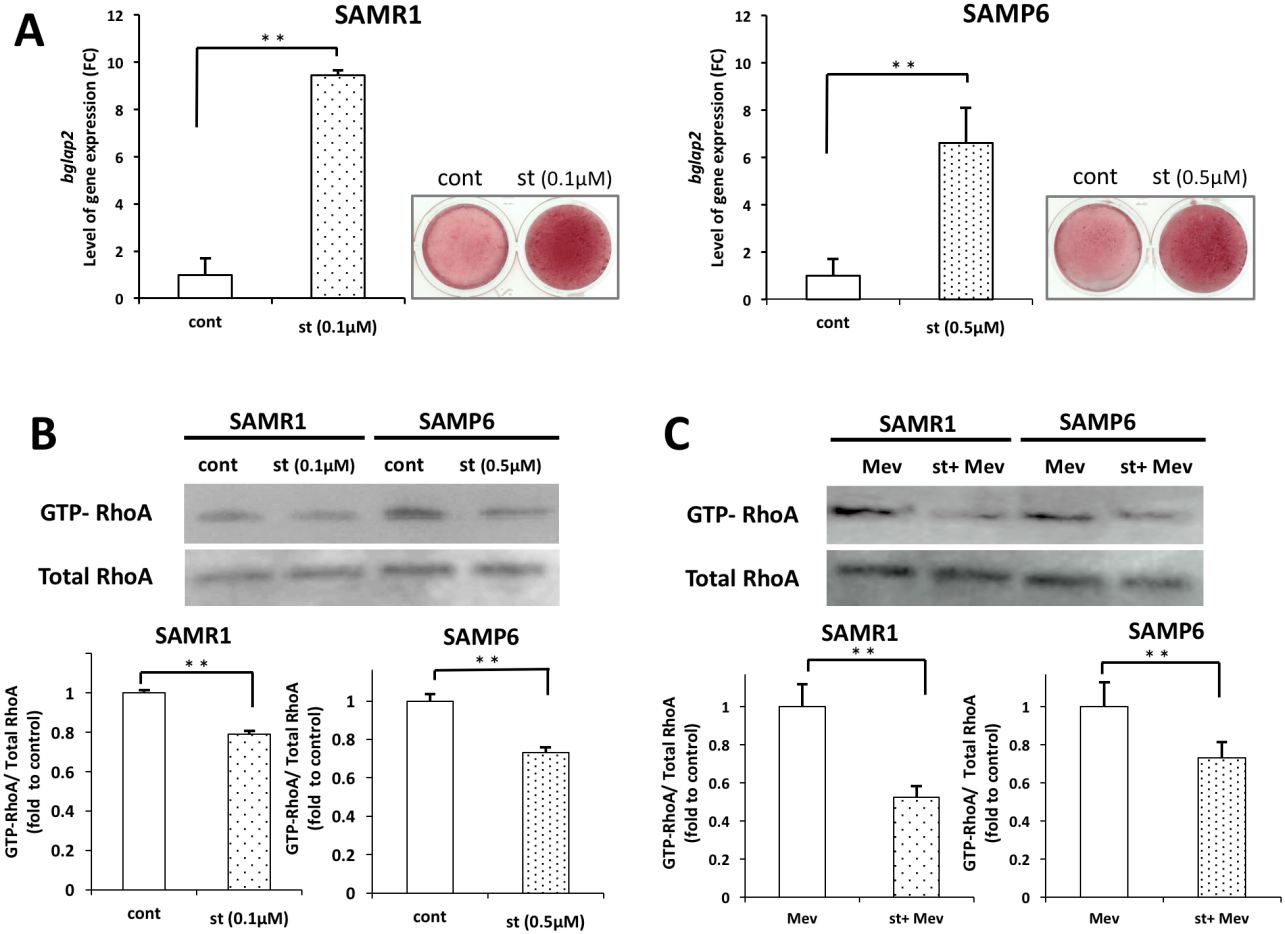
Mineralization and RhoA activation in SAMR1 and SAMP6 BMSCs. (A) SAMR1 and SAMP6 BMSCs were treated for 21days in the presence and absence of fluvastatin (SAMR1 at 0.1μM and SAMP6 at 0.5μM). The expression level of bglap2 mRNA was shown as FC (fold chnage), and mineralized matrix was visualized by Alizarin Red staining. (B) The level of GTP-bound RhoA was normalized by total RhoA shown as fold to control. SAMP6 and SAMR1 BMSCs were treated for 3days in the presence or absence of fluvastatin (SAMR1 at 0.1μM and SAMP6 at 0.5μM). (C) SAMP6 and SAMR1 BMSCs were treated with mevalonate for 3days in the presence and absence of fluvastatin (SAMR1 at 0.1μM and SAMP6 at 0.5μM). **P < 0.01.

Effects of fluvastatin on *bglap2* mRNA and protein expression detected with alizarin red staining are shown in Fig 5A. The *bglap2* mRNA expression in SAMR1 BMSCs treated with 0.1 μM, fluvastatin significantly increased compared with that in controls at 21 days, and *bglap2* expression significantly increased in SAMP6 BMSCs treated with 0.5 μM fluvastatin compared with controls at 21 days. In addition, mineralization in SAMR1 and SAMP6 BMSCs was highly observed at the presence of 0.1 and 0.5 μM fluvastatin.

### Effects of fluvastatin on RhoA activation

The activity of RhoA by rhotekin beads in SAMP6 and SAMR1 BMSCs was measured (Fig 5B). The level of GTP-bound RhoA was decreased in the presence of fluvastatin (SAMR1 at 0.1μM and SAMP6 at 0.5μM). The activity of RhoA in add-back experiments with mevalonate in the presence or absence of fluvastatin (SAMR1 at 0.1μM and SAMP6 at 0.5μM) (Fig 5C). The level of GTP-bound RhoA was decreased in the presence of fluvastatin.

## Discussion

Fluvastatin was used among several types of statins in this study. Based on their characteristics, statins can be classified into hydrophobic and hydrophilic subgroups. Hydrophobic statins include simvastatin and atorvastatin; hydrophilic statins include rosuvastatin and pravastatin. Hydrophobic statins cross the cellular membrane to enter cells, whereas hydrophilic statins rely on specific carrier mechanisms for entry into cells [15]. In contrast, fluvastatin appears to have intermediate physicochemical characteristics with both hydrophobicity and hydrophilicity because it is approximately twice as hydrophilic as lovastatin but 40 times more lipophilic than pravastatin [16]. Therefore, fluvastatin is considered to be suitable for cell culture because it is easily dissolved in the culture medium. Some authors suggested the positive effect of locally applied fluvastatin on bone healing [10]. Low-turnover osteoporosis, which is observed in both men and women, results in a decrease in the thickness of both cortical and trabecular bone due to osteoblastic hypoplasia [3,17]. Therefore, from a dental implant treatment perspective, it is important to improve bone healing for osseointegration in low-turnover osteoporosis. Several studies have used SAMP6 as models of low-turnover osteoporosis. In SAMP6 aged 16–20 weeks, reduced BMD, bone calcium, and bone phosphorous; decreased femoral weight and amount of trabecular bone because of osteoblastic hypoplasia; and thinning of cortical bone have been reported [18]. Therefore, in this study, SAMP6 were considered suitable for an experimental animal model of low-turnover osteoporosis.

The proliferation ability in SAMP6 tend to be lower than in SAMR1. A previous report confirmed that the BMSC proliferation ability in SAMR1 was significantly higher compared with SAMP6 [19]. No significant differences in cell proliferation were recognized among fluvastatin concentrations within 0–1.0 μM in SAMR1 or SAMP6 in this study. Similarly, Zhou et al.[20] demonstrated that concentrations of 0–1.0 μM simvastatin did not show any apparent inhibition of human adipose-derived stromal cells (hADSCs) proliferation; however, cell growth was inhibited at concentrations >1.0 μM. In addition, it was suggested that simvastatin concentrations >1.0 μM had a cytotoxic effect on human mesenchymal stem cells (hMSCs), because of cell death [21]. In our primary study, cell growth was also inhibited at 2.0 μM in SAMP6 and SAMR1 (data not shown). Accordingly, 0–1.0 μM fluvastatin were believed not inhibit cell proliferation of SAMP6 and SAMR1.

In the present study, differentiation of BMSCs into osteoblasts was investigated through the expression of osteoblast-associated genes (*bmp2*, *runx2* and *bglap2*), protein and endogenous ALP enzyme activity. Enhanced BMP2 expression for both gene and protein was observed in SAMP6 and SAMR1. Enhanced BMP2 expression was reported on simvastatin administration in human MG-63 osteoblasts [11,22], MC3T3-E1 cells [23] and normal mouse BMSCs [24]. Enhanced BMP2 expression was confirmed on fluvastatin administration in hMSCs [25]. In addition, we performed a BMP inhibition assay using noggin, a BMP-specific antagonist. As evidenced by histochemical analysis, noggin treatment significantly inhibited the BMP2-induced upregulation of ALP activity in SAMP6 and SAMR1. These results indicated that BMP2 expression was definitely increased by fluvastatin administration in SAMP6 and SAMR1 BMSCs.

Furthermore, it was reported that lipophilic statins crossed the cellular membrane [15] and increased BMP2 expression by inhibiting isoprenylation of Ras [26] and Rho [27] proteins in the mevalonate pathway. Ras/Rho proteins may be present in an inactive guanosine diphosphate (GDP)-bound cytosolic form, and upon cellular activation, they translocate to the active guanosine triphosphate (GTP)-bound membrane form. Ras/Rho proteins are dependent on isoprenylation for their activity [28]. Also in this study, the inhibition of RhoA activation was observed in the presence of fluvastatin in both SAMP6 and SAMR1. This result indicated that the BMP2 expression was increased by the inhibition of RhoA activation. Then, add-back experiments of the mevalonate pathway intermediates were also performed. By adding-back mevalonate, the RhoA inactivation was observed in the presence of fluvastatin in both SAMP6 and SAMR1. It suggested that mevalonate was involved in the resistance to fluvastatin in SAMP6. It was reported that fluvastain inhibits HMG-CoA reductase, an early step in the mevalonate pathway [9]. Thus, in the SAMP6 model, fluvastatin inhibited HMG-CoA reductase to reduce cholesterol production by mevalonate pathway but it also inhibited prenylation of proteins via geranylgeranylation.

Runx2 expression, ALP activity and mineralization were enhanced by fluvastatin treatment in SAMP6, in this study. It has been suggested that BMP2 activates Runx2 and regulates the ALP expression [29]. In addition, it was reported that enhanced *bmp2* mRNA expression by statins is a trigger of osteoblast differentiation because simvastatin induces the production of ALP in mouse osteoblasts [30]. Thus, in this study, fluvastatin stimulated differentiation of BMSCs into osteoblasts as a consequence of upregulated BMP2 and Runx2 expression and induced production of ALP and mineralization in SAMP6 BMSCs.

In the present study, BMP2 and Runx2 expression and ALP activity were increased at fluvastatin concentrations more than 0.5 μM in SAMP6, whereas BMP2 and Runx2 expression and ALP activity were increased at concentrations more than 0.1 μM in SAMR1. Accordingly, the minimum concentration of fluvastatin required to promote the differentiation of BMSCs into osteoblasts was 0.5 μM in SAMP6 and 0.1 μM in SAMR1. Previous studies indicated that the number of adherent colonies containing adipocytes was 14.7 times higher in SAMP6 BMSCs than in SAMR1 BMSCs and that the number of adipocytes was 5 times higher in SAMP6 than in SAMR1 [7]. In addition, in histological analysis, more adipose tissue was observed in SAMP6 [13]. Furthermore, it was reported that lipophilic statins such as simvastatin were transported into adipocytic cells [24]. Accordingly, a higher dose of fluvastatin is required against SAMP6 BMSCs to promote the differentiation into osteoblasts. In addition, it was demonstrated that osteoblastogenesis was decreased in SAMP6 compared with that in SAMR1. Thus, BMP2 and Runx2 expression and ALP activity is lower in SAMP6 than in SAMR1 when the same dose of fluvastatin is administered.

From our results, *runx2* mRNA expression in SAMP6 was delayed compared with that in SAMR1 and ALP activity in SAMP6 was lower than in SAMR1. It has been reported that higher expression levels of Sfrp4 negatively regulated bone formation and decreased BMD through inhibition of Wnt signaling in SAMP6 [31]. Therefore, our finding may be associated with this inhibition of Wnt signaling; however, the mechanism underlying the faulty differentiation of BMSCs into osteoblasts in SAMP6 remains unclear.

In this study, fluvastatin promoted osteoblastic differentiation mediated by RhoA-BMP2 action in SAMP6. Accordingly, the present result suggested that fluvastatin compensated for the decreased ability of BMSCs to differentiate into osteoblasts because of faulty Wnt signaling with promoted differentiation ability mediated by RhoA-BMP2 action in SAMP6. In mevalonate pathway, fluvastatin inhibited HMG-CoA reductase and prenylation of RhoA in low-turnover osteoporosis.

Fluvastatin may be clinically effective in terms of improving bone healing. With regard to the dose of fluvastatin, higher concentrations may be necessary in low-turnover osteoporosis cases than in normal cases. In addition, it may be effective to use local administration with a fluvastatin-releasing system in bone healing. This concept is supported by previous reports suggesting that local administration of fluvastatin facilitated bone formation in SAMP6 [13].

In conclusion, the present study revealed that fluvastatin promoted BMSC differentiation into osteoblasts by RhoA-BMP2 pathway in SAMP6. The concentration of fluvastatin for promoting BMSCs differentiation into osteoblast was higher in SAMP6 (low-turnover osteoporosis model mice) than in SAMR1 (control mice).

## References

[1] Riggs BL, Khosla S, Melton LJ, A unitary model for involutional osteoporosis: estrogen deficiency causes both type I and type II osteoporosis in postmenopausal women and contributes to bone loss in aging men. J Bone Miner Res 13 (1998) 763–73. 10.1359/jbmr.1998.13.5.763 9610739

[2] Alsaadi G., Quirynen M., Komárek A., & Van Steenberghe D. (2007). Impact of local and systemic factors on the incidence of oral implant failures, up to abutment connection. *Journal of Clinical Periodontology*, 34(7), 610–617. 10.1111/j.1600-051X.2007.01077.x 17433044

[3] Bryant S. R., & Zarb G. A. (2002). Outcomes of implant prosthodontic treatment in older adults. *Journal (Canadian Dental Association)*, 68(2), 97–102. 11869499

[4] Duque G, Troen BR, Understanding the mechanisms of senile osteoporosis: new facts for a major geriatric syndrome. J Am Geriatr Soc 56 (2008) 935–41. 10.1111/j.1532-5415.2008.01764.x 18454751

[5] Takeda T. Senescence-accelerated mouse (SAM): a biogerontological resource in aging research, Neurobiol Aging 20 (1999) 105–10. 10537019 10.1016/s0197-4580(99)00008-1

[6] Silva MJ, Brodt MD, Fan Z, Rho J-Y, Nanoindentation and whole-bone bending estimates of material properties in bones from the senescence accelerated mouse SAMP6. J Biomech 37 (2004) 1639–46. 10.1016/j.jbiomech.2004.02.018 15388305

[7] Kajkenova O, Lecka-Czernik B, Gubrij I, Hauser SP, Takahashi K, Parfitt AM, et al, Increased adipogenesis and myelopoiesis in the bone marrow of SAMP6, a murine model of defective osteoblastogenesis and low turnover osteopenia. J Bone Miner Res 12 (1997) 1772–9. 10.1359/jbmr.1997.12.11.1772 9383681

[8] Beppu K, Kido H, Watazu A, Teraoka K, Matsuura M, Peri-implant bone density in senile osteoporosis-changes from implant placement to osseointegration. Clin Implant Dent Relat Res 15 (2013) 217–26. 10.1111/j.1708-8208.2011.00350.x 21599831

[9] Shao H, Tan Y, Eton D, Yang Z, Uberti MG, Li S et al, Statin and stromal cell-derived factor-1 additively promote angiogenesis by enhancement of progenitor cells incorporation into new vessels. Stem Cells 26 (2008) 1376–84. 10.1634/stemcells.2007-0785 18308946

[10] Moriyama Y, Ayukawa Y, Ogino Y, Atsuta I, Koyano K, Topical application of statin affects bone healing around implants. Clin Oral Implants Res 19 (2008) 600–5. 10.1111/j.1600-0501.2007.01508.x 18422989

[11] Mundy G, Stimulation of Bone Formation in Vitro and in Rodents by Statins. Science (80-) 286 (1999) 1946–9. 10.1126/science.286.5446.1946 10583956

[12] Fukui T, Ii M, Shoji T, Matsumoto T, Mifune Y, Kawakami Y et al, Therapeutic effect of local administration of low-dose simvastatin-conjugated gelatin hydrogel for fracture healing. J Bone Miner Res 27 (2012) 1118–31. 10.1002/jbmr.1558 22275312

[13] Ohira T, Tanabe K, Sasaki H, Yoshinari M, Yajima Y, Original Effect of Locally Applied Fluvastatin in Low-turnover Osteoporosis Model Mouse with Femur Bone Defect. J Hard Tissue Bio 24 (2015) 147–54.

[14] Burnette W. N., "Western Blotting": Electrophoretic Transfer of Proteins from Sodium Dodecyl Sulfate-Polyacrylamide Gels to Unmodified Nitrocellulose and Radiographic Detection with Antibody and Radioiodinated Protein A, Analytical Biochemistry 112 (1981) 195–203. 6266278 10.1016/0003-2697(81)90281-5

[15] Horiuchi N, Maeda T, Statins and bone metabolism. Oral Dis 12 (2006) 85–101. 10.1111/j.1601-0825.2005.01172.x 16476028

[16] Hamelin BA, Turgeon J, Hydrophilicity/lipophilicity: relevance for the pharmacology and clinical effects of HMG-CoA reductase inhibitors. Trends Pharmacol Sci 19 (1998) 26–37. 9509899 10.1016/s0165-6147(97)01147-4

[17] Parfitt AM, Villanueva AR, Foldes J, Rao DS, Relations between histologic indices of bone formation: implications for the pathogenesis of spinal osteoporosis. J Bone Miner Res 10 (1995) 466–73. 10.1002/jbmr.5650100319 7785469

[18] Kasai S, Shimizu M, Matsumura T, Okudaira S, Matsushita M, Tsuboyama T, et al, Consistency of low bone density across bone sites in SAMP6 laboratory mice. J Bone Miner Metab 22 (2004) 207–14. 10.1007/s00774-003-0471-1 15108062

[19] O’Sullivan RP, Greenberger JS, Goff J, Cao S, Kingston KA, Zhou S, et al, Dysregulated in vitro hematopoiesis, radiosensitivity, proliferation, and osteoblastogenesis with marrow from SAMP6 mice. Exp Hematol 40 (2012) 499–509. 10.1016/j.exphem.2012.01.019 22326715 PMC3353019

[20] Zhou Y, Ni Y, Liu Y, Zeng B, Xu Y, Ge W, The role of simvastatin in the osteogenesis of injectable tissue-engineered bone based on human adipose-derived stromal cells and platelet-rich plasma. Biomaterials 31 (2010) 5325–35. 10.1016/j.biomaterials.2010.03.037 20381859

[21] Kupcsik L, Meurya T, Flury M, Stoddart M, Alini M, Statin-induced calcification in human mesenchymal stem cells is cell death related. J Cell Mol Med 13 (2009) 4465–73. 10.1111/j.1582-4934.2008.00545.x 19602044 PMC4515062

[22] Ruiz-Gaspa S, Nogues X, Enjuanes A, Monllau JC, Blanch J, Carreras R, et al, Simvastatin and atorvastatin enhance gene expression of collagen type 1 and osteocalcin in primary human osteoblasts and MG-63 cultures. J Cell Biochem 101 (2007) 1430–8. 10.1002/jcb.21259 17252541

[23] Maeda T, Matsunuma A, Kurahashi I, Yanagawa T, Yoshida H, Horiuchi N, Induction of osteoblast differentiation indices by statins in MC3T3-E1 cells. J Cell Biochem 92 (2004) 458–71. 10.1002/jcb.20074 15156558

[24] Song C, Guo Z, Ma Q, Chen Z, Liu Z, Jia H et al, Simvastatin induces osteoblastic differentiation and inhibits adipocytic differentiation in mouse bone marrow stromal cells. Biochem Biophys Res Commun 308 (2003) 458–62. 12914771 10.1016/s0006-291x(03)01408-6

[25] Benoit DSW, Nuttelman CR, Collins SD, Anseth KS, Synthesis and characterization of a fluvastatin-releasing hydrogel delivery system to modulate hMSC differentiation and function for bone regeneration. Biomaterials 27 (2006) 6102–10. 10.1016/j.biomaterials.2006.06.031 16860387

[26] Ghosh-Choudhury N, Mandal CC, Choudhury GG, Statin-induced Ras activation integrates the phosphatidylinositol 3-kinase signal to Akt and MAPK for bone morphogenetic protein-2 expression in osteoblast differentiation. J Biol Chem 282 (2007) 4983–93. 10.1074/jbc.M606706200 17179158

[27] Ohnaka K, Shimoda S, Nawata H, Shimokawa H, Kaibuchi K, Iwamoto Y, et al, Pitavastatin enhanced BMP-2 and osteocalcin expression by inhibition of Rho-associated kinase in human osteoblasts. Biochem Biophys Res Commun 287 (2001) 337–42. 10.1006/bbrc.2001.5597 11554731

[28] Auer J, Berent R, Weber T, Eber B, Clinical significance of pleiotropic effects of statins: lipid reduction and beyond. Curr Med Chem 9 (2002) 1831–50. 12369881 10.2174/0929867023369024

[29] Yamashita M, Otsuka F, Mukai T, Otani H, Inagaki K, Miyoshi T et al, Simvastatin antagonizes tumor necrosis factor-alpha inhibition of bone morphogenetic proteins-2-induced osteoblast differentiation by regulating Smad signaling and Ras/Rho-mitogen-activated protein kinase pathway. J Endocrinol 196 (2008) 601–13. 10.1677/JOE-07-0532 18310456

[30] Chen P-Y, Sun J-S, Tsuang Y-H, Chen M-H, Weng P-W, Lin F-H, Simvastatin promotes osteoblast viability and differentiation via Ras/Smad/Erk/BMP-2 signaling pathway. Nutr Res 30 (2010) 191–9. 10.1016/j.nutres.2010.03.004 20417880

[31] Nakanishi R, Akiyama H, Kimura H, Otsuki B, Shimizu M, Tsuboyama T et al, Osteoblast-targeted expression of Sfrp4 in mice results in low bone mass. J Bone Miner Res 23 (2008) 271–7. 10.1359/jbmr.071007 17907918

